# Variability of staffing and staff mix across acute care units in Alberta, Canada

**DOI:** 10.1186/s12960-016-0172-1

**Published:** 2016-12-01

**Authors:** Krishna Sharma, Stephanie E. Hastings, Esther Suter, Judy Bloom

**Affiliations:** Alberta Health Services, 10301 Southport Lane SW, Calgary, Alberta T2W 1S7 Canada

**Keywords:** Staffing levels, Staff mix, Nursing staff mix, Healthcare providers, Variability

## Abstract

**Background:**

The health workforce has a crucial position in healthcare, and effective distribution of the workforce is one of the critical areas for healthcare improvement. This requires a proper understanding of the allocation of healthcare providers including staffing levels and staffing variability within a healthcare system. High variability may imply significant differences in outcomes and greater opportunity to better distribute staffing and improve patient outcomes. The objective of this study was to examine staffing variation across acute care units in a large and integrated healthcare system.

**Methods:**

We used survey and administrative data on full time equivalencies of Registered Nurses, Licensed Practical Nurses, Health Care Aides, and allied health staff for 287 acute care units to examine staffing levels across multiple unit types. We used a subsample of 157 units in a more detailed analysis of staffing levels and staff distribution.

**Results:**

Results from the full sample indicate that staffing levels, particularly for Registered Nurses, vary substantially across unit types. Subsample analyses showed that the highest variation in staffing levels occurred in rural units, which also had higher average staffing for licensed practical nurses and allied health staff. Rural units had fewer Health Care Aides than did other units. The majority of units were staffed with a combination of all three nursing providers, but the most common arrangement in rural units was staffing of Registered Nurses and Licensed Practical Nurses only. We also found that units with the highest number Registered Nurses also tended to have higher numbers of other staff, particularly allied health providers.

**Conclusions:**

We observed significant variation in staffing levels and mix in acute care units. Some of the differences might be attributable to differences in patient needs and unit types. However, we also observed high variability in units with similar services and patient populations. As other research has shown that staffing is linked to differences in patient outcomes, there is an important opportunity to improve staffing for greater efficiency and higher quality care.

**Electronic supplementary material:**

The online version of this article (doi:10.1186/s12960-016-0172-1) contains supplementary material, which is available to authorized users.

## Background

Health workforce has a crucial position in healthcare as it serves as a vehicle for change to ensure quality health services. Accounting for more than 60% of the cost of providing inpatient care [1], the health workforce is also crucial for the sustainability of any healthcare system. Staffing has been a research interest since the early 2000s, and many studies in this area have demonstrated that staffing is strongly linked to important patient outcomes, such as inhospital mortality and adverse events [2–6]. Population needs-based planning involves using relative levels of need for care in various populations to determine how health human resources should be distributed, yet research shows that this planning is lacking in important ways [7]. Planning is typically based on utilization patterns or fiscal concerns rather than population needs [8], and the most common health human resources planning strategies do not account for the most important factors determining need [9].

How to create cost-effective staffing models that balance patient and population needs, workforce supply, and healthcare cost is a complex issue that sparks ongoing debate. Ideally, staffing (i.e., number and types of providers) is based on the need to ensure that we have the healthcare providers with the right skills, education, and experience to best fit patient needs. Context also plays an important role as workforce supply typically varies across geographic areas (e.g., urban/rural). Indeed, a Canadian government report noted that the supply of health professionals varies across the country and that these differences are likely explained in part by population health, but that other factors are at play [10]. Further complicating the matter is how staff are being deployed (the roles they have and how they enact their roles) and the model of care they work in (e.g., primary nursing vs. collaborative practice models) [11, 12].

Due to the lack of clear rules or guidelines to determine appropriate staffing on hospital units, it is difficult to determine staffing levels that are optimal for a given context. It is therefore likely that at least some staffing decisions are made on an ad hoc basis, resulting in variations in staffing even across similar units. Most studies linking staffing with patient outcomes, however, did not report on staffing variation—a measure of staffing differences across units or over time. The variation can be in staffing levels (the amounts of staff) or staff mix (the combination of different healthcare providers). While many studies in the past linked the variation in staffing models and intensity to variation in various outcomes in acute care settings [2–4, 13], very few studies focus on the extent and nature of staffing variability. To our knowledge, no studies report on staffing variability across hospital units in the Canadian context; the Canadian government has called for better understanding of whether healthcare systems have “the right mix of providers in the right places to keep patients close to home” [10]. Before healthcare systems can move forward with population needs-based planning, they must better understand the status quo of current staffing variability. High variability may imply significant differences in outcomes and greater opportunity to better distribute staff according to patient and population needs to improve patient outcomes.

The main objective of this study was to examine staffing variation across acute care units in a large single healthcare system serving the needs of an entire province. Specifically, we wanted to measure the staffing levels and mix of different non-physician care providers across a range of different acute care units in Alberta, Canada. We included three types of nursing-related providers—Registered Nurses (RNs), Licensed Practical Nurses (LPNs), and Health Care Aides (HCAs)—and allied health (AH) providers. RNs and LPNs are regulated nursing providers (that is, they are governed by a regulatory body mandated to protect and serve the public interest [14]), while HCAs are unregulated staff serving as aides to RNs and LPNs. RNs typically complete a 4-year degree program and may focus on higher acuity patients, whereas LPNs complete a 2-year diploma program and may be assigned to more stable patients, although the job duties often overlap considerably [15, 16]. AH providers are also regulated.

Alberta has a publicly funded healthcare system, and Alberta Health Services (AHS) is responsible for delivering health services to its over four million residents. Established by integrating 12 separate health entities in 2008, AHS is also Canada’s largest province-wide health system. With 106 acute care hospitals and 8471 hospital beds, AHS’s workforce exceeds 100 000 employees. Rising costs and overall demand to improve quality and safety have placed increasing pressure on healthcare organizations including AHS. One way to meet these demands is to improve how regulated and unregulated healthcare providers are deployed to achieve high-quality patient care [17], that is, ensuring that the right provider provides the right service at the right time. Since 2008, AHS has made great strides in integrating human resources and finance functions across the system, offering a unique opportunity to examine staffing patterns across a large-scale system.

## Methods

All acute care units (*n* = 440) in Alberta hospitals that provided inpatient care were eligible for this study. Hospital units eligible for inclusion were in hospitals ranging from large urban tertiary care facilities to small single unit rural hospitals. Units that were part of hospitals that only provided rehabilitation or long-term care were excluded from the survey. We used survey and administrative data for the last 3 months of 2013, which was also our study period.

### Data sources

#### Survey data

We sent online surveys to managers on the eligible units to collect staffing information that was not otherwise available from administrative data sources. In the survey, we listed all types of regulated and unregulated healthcare providers that units employed. This list included three types of nursing-related providers and 10 types of allied health (AH) providers. Nursing-related providers included only those who provided direct patient care including RNs, LPNs, and HCAs. AH providers included dietitians, social workers, therapists (physio, recreational, occupational, aides), pharmacists, pharmacy aides, speech language pathologists, and psychologists. For each provider type, we asked unit managers to provide their full time equivalents (FTEs). An FTE is equal to 1.0 if a staff member works full time, i.e., 37.75 h per week. The FTE value changes proportionally as hours are increased or decreased (e.g., a 0.5 FTE represents a staff member working just under 19 h per week). While data indicating FTE status were collected for each AH provider type, a single variable indicating combined FTE for all AH provider types was used in the analysis.

#### Administrative data

We used administrative data from the AHS Data Repository and Reporting System through the Data Integration Management and Reporting department. While the survey data was the source of staffing information, the administrative data provided patient information. We used patient data from administrative data sources to obtain information on patient flow in units. This was expressed in terms of number of patient days per month as a common denominator across units. We aggregated both staff and patient data at the unit level and linked them using unit and hospital identifiers.

Our main variable of interest was the provider FTEs per 100 patient days in a month where the monthly value was based on the average for the 3 months of our study period. This or a slight variant of this approach is commonly used in other studies to measure staffing that is adjusted for patient volumes [18]. We used the type of unit (e.g., medical, surgical, labor, and delivery) and the rural or urban setting of units as our classifying variables. The type of unit was based on information collected in the survey. However, the rural-urban classification of units was based on hospital designation determined by AHS based on the size of the community.

### Analysis

We first analyzed the data from the full study sample and reported basic descriptive statistics. We calculated average staffing levels of all nursing and allied health providers included in the study by unit types (e.g., medical, surgical, rural). We also created comparative charts describing staffing levels, mix, and variation by all unit types.

#### Study subsample

We used only a subsample of the data including urban medical, urban surgical, and rural units for a more detailed analysis. Urban units were general medical or surgical units whereas rural units were mixed units within rural hospitals providing multiple services including medical, surgical, obstetrics, and mental health services. The reason for selecting only these unit types was because these three types were among the most frequent units in the data which increased statistical rigor in the analysis. Within each unit type, units were assumed to have reasonably similar patient populations (e.g., rural units all serve fairly similar patients).

We analyzed the study subsample using distribution plots and quintile statistics. In the latter, we defined the quintiles by the staffing levels of a provider. This involved dividing the units into five equal-sized groups based on the ranking of proportions of RNs such that quintile 1 falls into the lowest 20% in the ranking (i.e., the lowest proportion of RNs relative to other providers), quintile 2 into 21 to 40%, and so on. Next, we calculated the average number of FTEs of RNs and other providers to compare average staffing of providers within and across those quintiles. Next, we calculated pairwise correlations between the staffing levels of all provider types for each unit type.

## Results

### Full sample

Of the total 440 units surveyed, 287 (64.5%) units returned the surveys. A few surveys had some missing data about AH provider FTEs. Of the total of 287 surveys included in the full sample, 23 (i.e., 8%) had at least one allied health FTE datapoint missing. In the analysis, all AH staffing numbers are based only on non-missing survey responses and all statistics are expressed in terms of monthly FTEs per 100 patient days.

#### Staffing levels

**Fig. 1:**
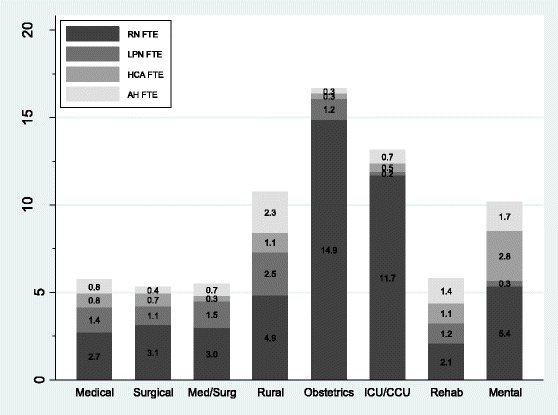
Average staffing of nursing and allied health providers across different unit types

Figure 1 shows the average FTEs per 100 patient days across all unit types (*N* = 287). The figure shows that staffing levels, particularly RN staffing, vary substantially across unit types. Unit types such as obstetrics and intensive/critical care units had substantially more RNs compared to other units, such as medical. In terms of staff mix, some units are more similar than others; medical, surgical, and medical/surgical units were fairly similar in terms of staffing levels and mix. On the other hand, rehab units have similar total staffing levels as medical and surgical units but their staff mixes were significantly different with much larger proportions of AH and HCA but smaller proportion of RN staffing. Table S1 (included as Additional file 1) presents detailed statistics, including the mean FTE of each provider by unit type, of all providers surveyed.

### Subsample

**Fig. 2:**
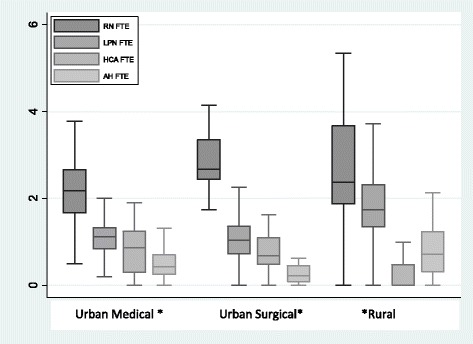
Box and whisker plots of staffing levels by unit types. *Note: For space outlier values are not shown

A total of 157 units met the selection criteria for the subsample analysis. Figure 2 shows the box plots of the overall staffing of all four provider groups by unit type for the subsample units. The plots show large variation of staffing levels of all providers across all units. The highest variation in staffing levels occurred in rural units which also saw higher average staffing levels of LPN and AH. Rural units also had the lowest staffing levels of HCA.

#### Distribution of staff mix

**Fig. 3:**
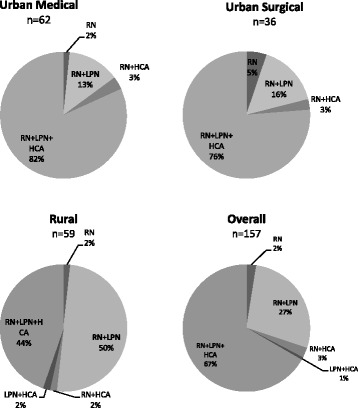
Prevalence of different staff mixes by unit types

For this analysis, we identified six different staffing arrangements: RN only, LPN only, RN and LPN only, RN and HCA only, LPN and HCA only, and RN, LPN, and HCA combined. We did not find any unit which was actually staffed with LPN only. The predominant way to staff units was through a combination of all three provider groups. The exception was in rural units, where the most prevalent arrangement was to staff with RN and LPN only (Fig. 3). This analysis also indicated that rural units had considerably less usage of HCAs in their staff mix.

#### Quintile analysis

We calculated the quintiles of the RN staffing levels for each unit type such that RN_1_ would include the units that fell into the first (i.e., bottom 20%) quintile of RN staffing, RN_2_ into the second quintile, and so on. Then for all five RN quintiles, we averaged the RN staffing as well as the LPN, HCA, and AH staffing. We repeated the quintile calculation for LPN, HCA, and AH and compared the results. To determine the statistical significance of the differences, we used one-way ANOVA tests. Table 1 shows these staffing quintiles to compare the levels and mix of different providers in urban medical, urban surgical, and rural units.

**Table 1:** Staffing levels and staff mixes by quintiles across major unit types

		Urban medical units (*n* = 62)							Urban surgical units (*n* = 36)						Rural units (*n* = 59)					
		1st	2nd	3rd	4th	5th	*p* value		1st	2nd	3rd	4th	5th	*p* value	1st	2nd	3rd	4th	5th	*p* value
Ranked by *RN* quintile	*RN*	*1.1*	*2.0* ^†^	*2.4* ^‡^	*2.9* ^‡^	*7.2* ^§^	*0.00*		*1.7*	*2.1*	*2.5*	*2.9* ^†^	*5.1* ^§^	*0.00*	*1.1*	*2.1*	*2.4*	*3.2*	*9.6* ^§^	*0.01*
	LPN	1.3	1.1	1.1	1.0	1.3			0.9	1.5	1.2	1.0	0.9		1.3	1.6	1.6	1.9	4.4	
	HCA	1.0	0.9	0.6	0.9	0.3			0.9	0.6	0.6	0.8	0.8		0.4	0.2	0.2	0.5	2.1	
	AH	0.4	0.5	0.4	0.4	3.7			1.1	0.4	0.2	0.2	0.7		0.5	0.7	0.9	0.9	4.2	
	*Total*	3.8	4.5	4.5	5.2	12.5			4.6	4.6	4.5	4.9	7.5		3.3	4.6	5.1	6.5	20.3	
Ranked by *LPN* quintile	RN	3.6	2.3	2.1	2.2	3.0			3.6	3.0	3.1	2.4	3.6		13.8	2.6	2.1	2.0	5.1	
	*LPN*	*0.4*	*1.0* ^§^	*1.3* ^§^	*1.6* ^§^	*2.8* ^§^	*0.00*		*0.5*	*1.0* ^§^	*1.3* ^§^	*1.6* ^§^	*2.9* ^§^	*0.00*	*0.4*	*1.0*	*1.3*	*1.6*	*3.9* ^‡^	*0.00*
	HCA	0.9	0.8	0.9	1.1	0.4			1.0	0.7	0.5	0.6	0.3		0.5	0.2	0.4	0.4	1.5	
	AH	1.8	0.5	0.5	0.4	0.6			0.3	0.6	0.3	0.2	0.2		2.1	0.6	0.7	0.8	2.9	
	*Total*	6.7	4.6	4.8	5.3	6.8			5.4	5.3	5.2	4.8	7		16.8	4.4	4.5	4.8	13.4	
Ranked by *HCA* quintile	RN	5.3	1.9	2.3	2.3	1.8			3.0	2.6	3.2	2.8	3.7		5.1	1.7	2.6	3.2	4.8	
	LPN	1.7	1.3	1.2	1.0	1.1			1.7	1.4	1.2	0.7	0.9		2.7	1.2	1.2	1.2	3.5	
	*HCA*	*0.0*	*0.3* ^‡^	*0.6* ^§^	*1.0* ^§^	*1.5* ^§^	*0.00*		*0.0*	*0.3* ^†^	*0.6* ^§^	*1.0* ^§^	*1.4* ^§^	*0.00*	*0.0*	*0.2*	*0.5*	*1.0*	*4.1* ^§^	*0.00*
	AH	2.8	0.4	0.5	0.5	0.5			0.3	0.0	0.3	0.5	0.4		2.2	0.6	0.8	0.3	1.6	
	*Total*	9.8	3.9	4.6	4.8	4.9			5	4.3	5.3	5	6.4		10	3.7	5.1	5.7	14	
Ranked by *AH* quintile	RN	2.0	2.1	2.0	2.3	6.7			2.5	3.2	3.6	2.4	4.0		2.8	1.8	2.9	2.6	6.9	
	LPN	1.2	1.1	0.8	1.6	0.9			1.2	1.3	0.7	1.4	0.8		2.3	1.2	1.9	1.9	3.2	
	HCA	0.7	0.9	1.2	0.8	0.5			0.6	0.8	0.8	0.0	0.9		0.5	0.3	0.4	0.2	1.7	
	*AH*	*0.0*	*0.3*	*0.4*	*0.7*	*4.3* ^§^		*0.00*	*0.0*	*0.2* ^§^	*0.4* ^§^	*0.6* ^§^	*1.4* ^§^	*0.00*	*0.0*	*0.3*	*0.4*	*0.8*	*3.9*	*0.20*
	*Total*	3.9	4.4	4.4	5.4	12.4			4.3	5.5	5.5	4.4	7.1		5.6	3.6	5.6	5.5	15.7	

All statistics are FTEs per 100 patient days

Italicized rows are the focal provider in each analysis

^†^ *p* value <0.05; ^‡^ *p* value <0.01; and ^§^ *p* value <0.001 in comparison with 1st quintile

The first RN quintile, which meant the units with the lowest 20% of RN staffing, had 1.1 mean RN FTEs per 100 patient days. In those units, the staffing of LPN, HCA, and AH were 1.3, 1.0, and 0.4 FTEs respectively. The *p* values reported provide overall fit test or *F*-test results from one-way ANOVA tests. Results reported in superscripts represent the *p* values of pairwise test results of the reported value in comparison with the reference value (first quintile in this case).

As Table 1 shows, the variations of staffing level and mix between quintiles were substantial within each unit type. Compared to the first quintile, the fifth quintile had more than six times as many RNs in urban medical units, three times in urban surgical units, and nine times in rural units. Even if we exclude two extreme quintiles, RN staffing levels on the fourth quintile were about 1.5 times the staffing levels of the second quintile on all unit types.

The pattern of RN staffing level and variation was very similar across all three unit types with the exception of the fifth quintile. The pattern was the same for LPN, HCA, and AH providers. A consistent pattern was that highly staffed units had high levels of staffing of all providers. This is particularly evident in the case of RN and allied health providers—units with high levels of RN staffing also had high allied health staffing. The difference was most striking among rural units where the fifth quintile was most highly staffed by all providers in comparison to other unit types and quintiles. Note that the *F*-statistics of all models were significant except for rural units ranked by AH, but not all quintile means were statistically significant in comparison with the means of baseline quintiles.

Table 2 presents correlation coefficients between pairs of four provider types by unit types. The results show negative correlations between RN and HCA on urban medical and between LPN and HCA on urban surgical units. However, the staffing of LPN and HCA was positively correlated on rural units. Similarly, AH staffing was positively correlated with RN staffing on all unit types and with LPN staffing on rural units. Although most correlations were small, they were statistically significant. Negative correlations may indicate some degree of substitution between different types of nursing providers, while positive correlations may indicate that some providers were complementary to others.

**Table 2:** Correlations of staffing levels across major unit types

Urban medical					Urban surgical				Rural			
	RN	LPN	HCA	AH	RN	LPN	HCA	AH	RN	LPN	HCA	AH
RN	1				1				1			
LPN	−0.12	1			−0.13	1			0.24	1		
HCA	−0.42*	−0.24	1		0.08	−0.45*	1		0.14	0.42*	1	
AH	0.74*	−0.19	−0.22	1	0.39*	−0.25	0.18	1	0.41*	0.79*	0.11	1

*Significant at *p* < .05

## Discussion

Finding the right staff mix is critically important in the changing landscape of healthcare delivery, resource constraints, workforce shortages, and changing patient needs in acute care settings. This study examined the staffing patterns in acute care units in a large provincial health authority. Any evidence of significant variation in staffing levels and staff mix would provide us with reason to investigate further if staffing variation could be linked to differences in patient outcomes.

Our analysis of the full study sample found that there was substantial variation in the staffing level and mix across acute care settings. This might be a reflection of the different services provided by the units. For example, intensive care and obstetrics units are staffed with more providers generally and with far more RNs per 100 patient days than are the other unit types; these units are also quite different in terms of the level and intensity of services. Staff mix was predictably quite different in those unit types compared to the others. Similarly, mental health units had more HCAs per 100 patient days than did any other unit type. Interestingly, our results showed lower AH staffing in rehabilitation units than in some other units (e.g., rural).

In our detailed analysis of AHS staffing, we examined the staffing levels and variation within a subsample of units classified as urban medical, urban surgical, and rural based on their services and/or location. Within each of these unit types, we would expect to have similar patient populations and, hence, similar staffing requirements. However, staffing level and mix within those unit types varied quite substantially. Variation in staff mix and staffing levels seemed to be driven by factors that also affected RN staffing; this could be the result of a “halo effect” where hospitals with good nurse staffing are generally well-resourced clinically [19]. Indeed, we found that units in the highest quintile of RN staffing typically also had substantially more of all types of providers relative to the other quintiles, particularly in rural units. Little research has examined RN staffing in conjunction with staffing of other providers [19], so it remains to be tested whether this increased staffing across the board is a cost-effective means of improving patient outcomes. That said, research shows that lower nurse-patient ratios (such as those in Magnet hospitals) can increase nurse satisfaction, decrease burnout, and reduce the likelihood of leaving a position [20], which may increase patient satisfaction [21].

In a few cases, we observed moderate negative correlations between the staffing levels of different nursing providers. There could be various factors to explain this phenomenon, but a part of the correlation could be due to the substitution of one provider type for another. The trade-offs between HCA and RN/LPN staffing to a certain degree could be the result of cost saving strategies, i.e., the substitution of lower cost providers observed elsewhere [22, 23]. AHS has, at various times, encouraged increased hiring of LPNs or HCAs in place of RNs due to economic constraints. This is also part of the drive for team nursing models, such as those where a small number of RNs oversee care for a larger number of other providers [24]. On the other hand, allied health staffing was more complementary to RN staffing [25]. The degree of substitution and complementation should be examined further in a more focused study.

Rural units were particularly different both in terms of staff mix and staffing levels. More rural units had RNs and LPNs in their staffing pool compared to urban units, which were more likely to have RNs, LPNs, and HCAs, and their overall staffing was much higher than those of urban medical and surgical units. This indicates that rural units have different staffing practices and that there might be a greater potential to optimize staffing by readjusting staffing levels and mixes in rural units. The reasons for this different staffing in AHS rural units are unknown; future research should consider whether the decisions are based on population needs, differences in availability of certain providers, or other factors.

High variability in staffing may have substantial impacts on patient care. For example, an American study found that staffing variation was strongly linked with differential patient outcomes [2]. In a large research study on nursing staffing commissioned by the Agency for Healthcare Research and Quality [26], researchers concluded that higher nurse staffing levels resulted in lower incidences of adverse events among patients. Specifically, “higher rates of RN staffing were associated with an [overall] 3- to 12-percent reduction in adverse outcomes, [and] higher staffing at all levels of nursing was associated with an [overall] 2- to 25-percent reduction in adverse outcomes” ([26], p. 3). Mandated licensed nurse-to-patient ratios, which would prescribe the minimum staffing levels required in a given unit, have gained momentum in some countries. A review of the literature revealed that the support for this approach is largely anecdotal, and there is little to no empirical evidence to support the finding [27]. In fact, a study of California’s mandated minimum nurse staffing levels found no “persuasive evidence” that the law improved patient safety [28]. Most healthcare staffing literature focuses on the effects of increasing or decreasing nurse staffing levels in different environments (e.g., acute care, community care, surgical units) [29, 30]. This information seems to be geared more towards assisting administrators in managing staffing levels in individual units, rather than in developing a standardized staffing ratio.

The high variability in staffing level and mix has major implications for human resources planning as well as overall staffing cost. We do not fully understand all the factors that drive staffing decisions, but it seems unlikely that the variability within unit types in AHS can be explained by population and/or patient acuity or needs alone. AHS is a large healthcare system with considerable diversity, consisting of many small and large urban, regional, and rural hospitals spread across a large geographical area. Given that AHS was amalgamated from previously distinct and independent regional authorities, different historical staffing practices and budget allocations may have contributed to this finding. These differences, when taken alongside the importance and underuse of population needs-based planning [7, 9], suggest that AHS has much work to do to ensure that the right providers are available for patients in the appropriate settings.

## Conclusions

We observed considerable variation in staffing levels and mix in acute care units. Some of the differences might be attributable to differences in patient needs and unit types providing different services. However, we also found unexpected variability in units with similar services and patient populations. As variation is linked to differences in patient outcomes, there may be an important opportunity to better distribute staffing for greater efficiency and higher quality care within AHS. Accordingly, future studies in this area should look at differences in patient outcomes between units with different staffing levels and staff mixes. Such studies should also explore in more detail how providers other than nurses factor into the complex relationship between staffing and patient outcomes, given our finding that allied health staffing tends to vary with staffing of other providers.

## Additional file

Additional file 1: Table S1. Mean (SD) statistics of all providers by unit service. (DOCX 17 kb)

## Abbreviations

AH: Allied health
AHS: Alberta Health Services
FTE: Full time equivalency
HCA: Health care aide
LPN: Licensed practical nurse
RN: Registered nurse
