# Supplementary material for: Variability of staffing and staff mix across acute care units in Alberta, Canada
Source: Hum Resour Health. 2016 Dec 1;14:74. doi: 10.1186/s12960-016-0172-1 (PMC5131495; doi:10.1186/s12960-016-0172-1)
Supplement: Additional file 1: Table S1. — Mean (SD) statistics of all providers by unit service. (DOCX 17 kb) [file 12960_2016_172_MOESM1_ESM.docx]

| Table S1: Mean (SD) statistics of all providers by unit service | | | | | | | | |
| --- | --- | --- | --- | --- | --- | --- | --- | --- |
|  | Medical  N=76 | Surgical  N=36 | Medical/ surgical  N=27 | Obstetric and newborn  N=23 | Rural – multiple services  N=33 | Special care ICU/CCU  N=26 | Rehab  N=18 | Mental health  N=48 |
| Registered Nurse | 2.73 (2.18) | 3.14 (1.2) | 3 (1.62) | 4.85 (9.02) | 14.88 (14.16) | 11.71 (8.09) | 2.08 (0.98) | 5.36 (16.09) |
| Licensed Practical Nurse | 1.43 (1.23) | 1.08 (0.64) | 1.49 (0.71) | 2.48 (3.1) | 1.2 (1.77) | 0.18 (0.57) | 1.17 (0.95) | 0.33 (0.48) |
| Health Care Aide | 0.8 (0.64) | 0.72 (0.44) | 0.33 (0.46) | 1.08 (3.15) | 0.32 (1.51) | 0.5 (0.84) | 1.15 (1.35) | 2.81 (10.14) |
| Social Worker | 0.14 (0.4) | 0.05 (0.07) | 0.09 (0.1) | 0.03 (0.05) | 0.17 (0.35) | 0.16 (0.21) | 0.23 (0.22) | 0.46 (1.01) |
| Dietician | 0.21 (1.19) | 0.04 (0.05) | 0.08 (0.09) | 0.2 (0.73) | 0.04 (0.07) | 0.12 (0.15) | 0.03 (0.04) | 0.05 (0.23) |
| Occupational Therapist | 0.18 (0.79) | 0.07 (0.12) | 0.08 (0.06) | 0.11 (0.16) | 0 (0.01) | 0.05 (0.08) | 0.25 (0.23) | 0.2 (0.5) |
| Physiotherapist | 0.09 (0.1) | 0.12 (0.17) | 0.11 (0.08) | 0.36 (1.16) | 0.01 (0.01) | 0.21 (0.33) | 0.28 (0.36) | 0.01 (0.04) |
| Therapy Aides | 0.06 (0.08) | 0.04 (0.08) | 0.07 (0.07) | 0.69 (2.28) | 0.01 (0.04) | 0.05 (0.15) | 0.25 (0.29) | 0.56 (2.51) |
| Pharmacists | 0.08 (0.09) | 0.04 (0.07) | 0.1 (0.1) | 0.41 (1.46) | 0.07 (0.19) | 0.23 (0.23) | 0.05 (0.07) | 0.05 (0.08) |
| Pharmacy Techs | 0.03 (0.09) | 0.01 (0.02) | 0.1 (0.18) | 0.52 (1.57) | 0.02 (0.06) | 0.02 (0.07) | 0.01 (0.02) | 0.01 (0.03) |
| Recreational Therapist | 0.02 (0.06) | 0.02 (0.06) | 0.03 (0.06) | 0.08 (0.15) | 0.02 (0.08) | 0 (0) | 0.12 (0.16) | 0.19 (0.5) |
| Speech Language Pathologist | 0.03 (0.06) | 0 (0.02) | 0.04 (0.05) | 0.03 (0.07) | 0 (0) | 0.02 (0.04) | 0.09 (0.16) | 0 (0.01) |
| Psychologist | 0.01 (0.09) | 0.39 (0.44) | 0 (0.02) | 0 (0.01) | 0 (0) | 0 (0.01) | 0.12 (0.26) | 0.22 (0.54) |
| All allied health | 0.87 (2.34) | 0.39 (0.44) | 0.69 (0.44) | 2.42 (7.05) | 0.34 (0.58) | 0.87 (0.93) | 1.42 (1.34) | 1.76 (4.93) |
| **Notes**:   - All values are expressed as FTE per 100 patient days - Zeros values are values <0.05 rounded to zero. | | | | | | | | |
